# Magnetic resonance imaging findings of intravascular leiomyomatosis of the uterus: a case report

**DOI:** 10.3389/fmed.2023.1328339

**Published:** 2024-01-24

**Authors:** Bin Guo, Shuhui Zhao, Juan Li, Xiaoyan Wang

**Affiliations:** ^1^Department of Radiology, Jinan Maternal and Child Care Hospital, Jinan, Shandong Province, China; ^2^Department of Gynecology, Jinan Maternal and Child Care Hospital, Jinan, Shandong Province, China; ^3^Department of Pathology, Jinan Maternal and Child Care Hospital, Jinan, Shandong Province, China

**Keywords:** intravascular leiomyomatosis, uterine leiomyoma, variant types of leiomyoma, benign metastasizing leiomyoma, misdiagnosis, MRI

## Abstract

**Background:**

Intravascular leiomyomatosis (IVL) is often a non-malignant condition that grows inside the blood vessels and does not invade surrounding tissues. However, its presence within the blood vessels can lead to obstructions. The majority of IVL cases manifest symptoms related to blockage.

**Case presentation:**

We present a case of a 50-year-old female patient who was referred to our institution due to the presence of a common non-cancerous tumor in the uterus, known as a benign uterine leiomyoma. The tumor was identified during an ultrasound examination during a routine physical examination. Postoperative pathology established the existence of intrapelvic IVL.

**Conclusion:**

Intrapelvic IVL often not present with complications when it is confined to the pelvic cavity. Furthermore, the imaging features of intrapelvic IVL resemble those of typical benign uterine leiomyomas. This can often result in the clinical misdiagnosis of the tumor as a typical benign uterine leiomyoma.

## Introduction

Intravascular leiomyomatosis (IVL) has the typical morphological and histological characteristics of a benign uterine leiomyoma, but it grows inside the blood vessels without invading the surrounding tissue. While the condition is benign, it has the potential to propagate via the pelvic veins to the right cardiac chambers, causing blockage. The typical age of onset ranges from 40 to 60 years, and around 60% of patients have a prior history of uterine myoma (1). IVL is recognized as a rare form of uterine myoma. The stage of intravenous leiomyomatosis was categorized into 4 stages helping surgical management (2, 3). Stage I is considered if the tumor involving the uterine vein was confined to the pelvic cavity. Stage II corresponds to a tumor extending into the abdominal cavity. If the tumor reaches the right atrium beyond the renal vein, it is categorized as stage III. Stage IV involves the invasion of tumors to the pulmonary artery and even the metastasis of tumors to the lungs (4). There may have been a reporting bias in the literature because a large number of reports have concentrated on extrapelvic IVL, maybe as a result of the presentation of obstruction-related symptoms (5). Moreover, as far as we know, there have been no documented cases of asymptomatic intrapelvic IVL being identified as an abnormality in medical examination results. In this report, we present a case of intrapelvic IVL and provide a comprehensive assessment of the existing literature on this condition. The imaging features are additionally supplied as a point of reference for clinical diagnosis and treatment.

The ultrasound and magnetic resonance imaging (MRI) features of intrapelvic IVL are similar to those of typical benign uterine leiomyomas. Nevertheless, IVL exhibits variations from conventional benign uterine leiomyomas. The treatment of typical benign uterine leiomyoma includes hysteromyomectomy or hysterectomy. Nevertheless, IVL is reliant on estrogen and tends to reoccur. Consequently, its treatment typically entails performing a total abdominal hysterectomy and bilateral oophorectomy, particularly in older individuals, to minimize the chances of recurrence. Additionally, efforts are made to eliminate as much of the extrauterine tumor as feasible. Recurrence can result from incomplete excision and may necessitate further surgical treatment.

Routine blood preparation before surgery may be insufficient as adequate blood preparation is necessary due to the tumor growth within venous channels. Understanding the imaging characteristics of intrapelvic IVL can be extremely helpful in a precise diagnosis. Timely surgical treatment and early diagnosis are essential for a successful outcome in this case. Treatment should not be delayed. Following surgery, the current case was monitored for 3 years without experiencing a recurrence.

## Case presentation

A 50-year-old perimenopausal woman was referred to our institution. The patient was asymptomatic, and the presence of a uterine leiomyoma had been detected by ultrasound during a physical check-up. The uterine lesion was initially diagnosed as a benign leiomyoma. She had a history of abortion but no previous operations nor a history of myoma or adenomyosis. The gynecological examination showed no abnormalities in the vulva and vagina, 2/3 erosion of the cervical surface, an anteriorly positioned uterus, no tenderness, increased volume, morphological under-regulation, and a 6 cm diameter cystic mass at the left rear side of the uterus. The mass was smooth, had defined limits, and moved properly without causing pain. There was no palpable bulk in either of the bilateral adnexal regions. Normal results were obtained from standard laboratory tests on the blood and urine. The serum concentrations of CA199 and CA125 were within normal ranges.

**Figure 1:**
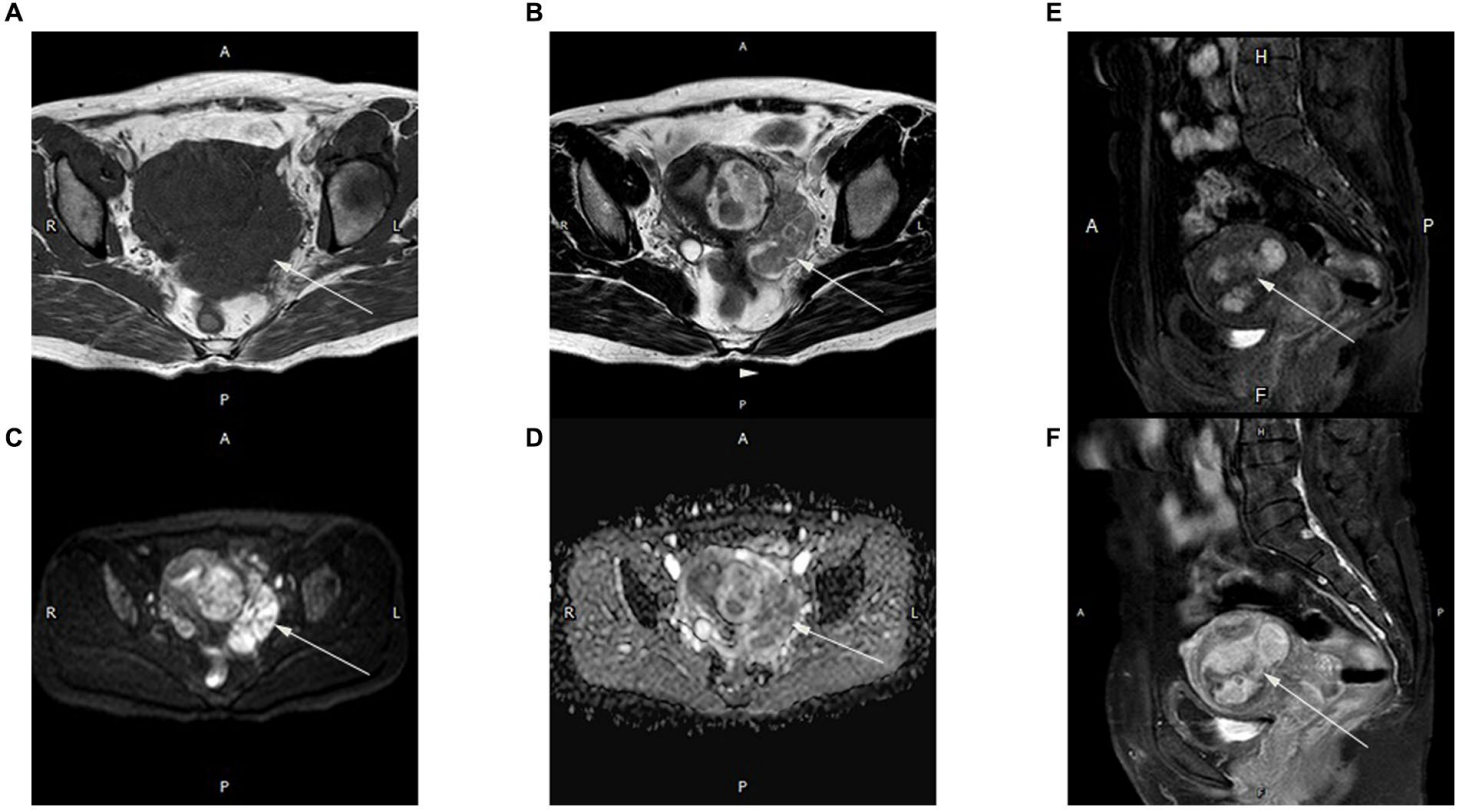
Magnetic resonance (MR) images of an intrapelvic IVL tumor in a 50-year-old woman. A mixed solid and cystic pelvic mass extended from the intermuscularis to the subserosa, as well as the extrauterine environment and had a worm-like appearance. The lesions showed isointensity on T1WI **(A)** and inhomogeneous signal intensity on T2WI **(B)** (arrow). The diffusion-weighted image (b-value = 800 s/mm^2^) **(C)** indicates high signal intensity in most of the mass (arrow). Apparent diffusion coefficient (ADC) mapping **(D)** shows high intensity within the mass (arrow). Strong homogeneous enhancement is seen in the arterial **(E)** and delayed **(F)** phases on the T1 sagittal image. All images were obtained using a 1.5-T MR scanner (Achieva, Philips Medical Systems).

Pelvic MRI confirmed a tumor mass of about 7.6 × 5.5 cm in size at the left posterior wall of the uterus and extending into the parametrium. The tumor exhibited a worm-like morphology and displayed an equivalent signal intensity on T1-weighted imaging (T1WI) and a non-uniform signal intensity on T2-weighted imaging (T2WI), along with a heightened signal on diffusion-weighted imaging (DWI) without any low apparent diffusion coefficient (ADC) value. Significant enhancement of the T1W1 signal was observed after gadolinium chelate injection (Figure 1). The endometrium was pressured, and the lymph nodes were normal.

**Figure 2:**
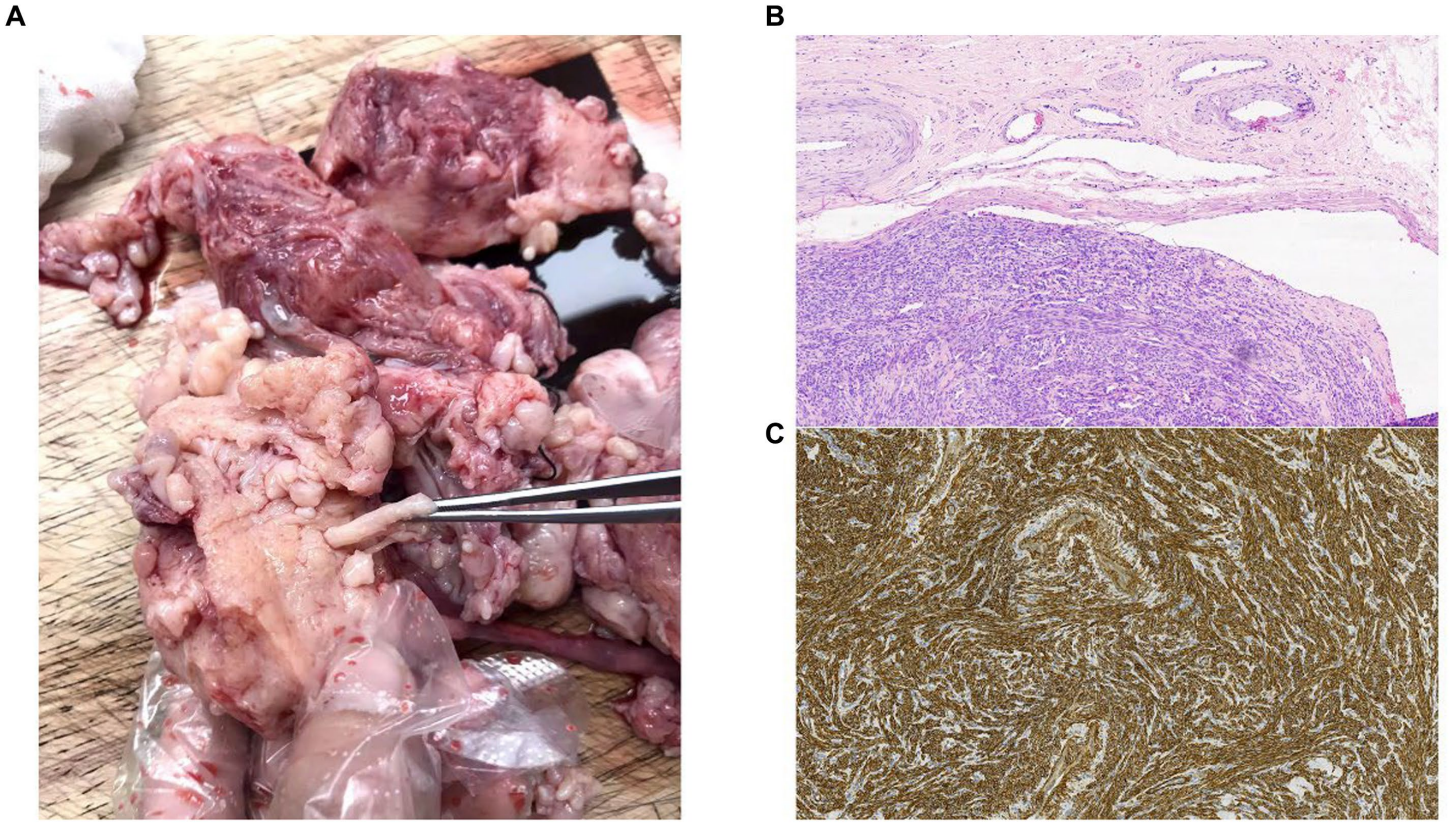
**(A)** Images from the operating theater. **(B)** Histology of the resected tumor at ×10 magnification. Intravascular tumor presents within the lumen of a vein, showing dense proliferation of uniform spindle-shaped smooth muscle cells. **(C)** The smooth muscle compartment is shown by h-caldesmon staining (h-caldesmon, 100×).

A total abdominal hysterectomy and bilateral salpingo-oophorectomy were performed. During the laparotomy, the myometrium exhibited many and subserosal fibroids and appeared nodular. The maximal diameter of the fibroids was approximately 3 mm, and worm-like structures were visible between the uterine fundus and cervix. Both the left uterine vein and the left wide ligament were involved. There were no apparent abnormalities of the fallopian tubes or ovaries. Pathological evaluation of the specimen confirmed the diagnosis of IVL (Figure 2).

## Discussion

The number of cases of extrapelvic IVL is higher in many reported studies, although intrapelvic IVL cases are uncommon. Through conducting a PubMed search with the specific keywords “intravenous leiomyomatosis,” we identified 2,338 studies. However, through conducting a PubMed search with the specific keywords “intrapelvic intravenous leiomyomatosis,” we identified two studies (6, 7). IVL is a benign tumor caused by the proliferation of smooth muscle cells within the vasculature. It is usually seen in the pelvic vein (uterine veins, ovarian vein, and iliac vein), inferior vena cava, renal vein, and pulmonary artery but can also metastasize to the heart, lung, brain, and lymph nodes (8–10). There is no invasion of the vascular tissue. The symptoms vary based on the tumor’s location and the extent of intravascular growth. Tumor cells can move to establish themselves in the pulmonary artery, resulting in symptoms such as chest pain, syncope, and dyspnea in extreme cases. When the lesions are confined to the pelvis, some patients do not have any clinical symptoms of asymptomatic or specific serologic tumor markers, leading to difficulties in early diagnosis and a high rate of missed diagnoses.

There are two perspectives regarding the development of IVL. One characteristic of uterine leiomyomatosis is that myometrial leiomyoma infiltrates into the venous system. The other pertains to the tumor arising directly from the walls of the vein (11). The majority of patients experience uterine leiomyoma, and Kir’s study revealed that intravascular tumor cells had progesterone receptor positivity ranging from weak (10%) to strong (70%), as well as estrogen receptor positivity ranging from weak (10%) to strong (60%). The endothelium and subendothelial cells exhibited insignificant progesterone and estrogen receptor positivity, which was very weak (12). These results do not support the hypothesis of a vessel wall origin for intravenous leiomyomatosis. Apart from its hormone responsiveness, little about the pathobiology of IVL, a rare smooth muscle proliferation, is known. Some research suggests (13, 14) that IVL arises from a uterine leiomyoma with a t (12; 14) (q15; q24). The presence of an extra copy of 12q15-qter and/or loss of 14q24-qter may be a critical genetic event(s) leading to intravascular intrusion and proliferation.

Differential diagnosis includes uterine leiomyoma, intrapelvic IVL and endometrial stromal sarcoma (ESS). Uterine leiomyoma is the most common myometrium tumor. ESS is the second most common type of uterine sarcoma (15). IVL can imitate endothelial cell sarcoma (ESS) due to the shared characteristic of both disorders spreading as a continuous tumor into the surrounding vascular systems. IVL and uterine sarcomas often occur with uterine leiomyoma, and this association may result in comparable clinical manifestations. The primary clinical signs included either no symptoms or the presence of a uterine tumor, abnormal uterine bleeding, and pelvic pain. The imaging features of IVL and ESS can resemble those of uterine leiomyoma. Both IVL and ESS are depicted as hypointensive signals on T2-weighted imaging. However, the uterine leiomyoma has even signals hypointensity on T2 weighted imaging, a regular margin with subsequent homogeneous enhancement. Nevertheless, both IVL and ESS indicate that the parametrial mass protrudes from the uterine mass, and its boundary is indistinct. The T2 weighted imaging shows heterogeneity, with a combination of hypointensive and hyper-intensive signals. Moreover, they appear characteristic “worm-like.” The parametrium and adnexa are invaded to various degrees. In addition, imaging of ESS reveals a hyperintensity on T1 weighted imaging, a significant hyperintensity on DWI, and a hypointensity on the apparent diffusion coefficient. The lesion of ESS has restricted diffusion in DWI. However, the imaging of IVL reveals that both diffusion-weighted imaging and apparent diffusion coefficient lesions display elevated signals with unrestricted diffusion. Moreover, a feature distinctive of sarcoma is high signal intensity (SI) (16). On T2WI, a hyper-intense signal suggests high cellularity or high vascularity of uterine sarcoma. The pathological high signal on T1 weighted imaging bases was related to hemorrhage, which suggests sarcoma of the uterus (17). Malignant lesions are more prone to hemorrhage in comparison to benign ones. DWI combined with T2WI significantly improves the possibility of distinguishing uterine sarcomas from benign leiomyomas, as has been shown by Namimoto et al. (18) DWI combined with T2WI demonstrated the highest sensitivity and the highest specificity, both estimated at 100%. At the early stage of IVL, most of the patients were asymptomatic, and the tumor extension remained inside the small vessels of the myometrium and could not be detected by CT scan or MRI. The presence of an apparently normal uterus does not preclude the possibility of IVL (19). Therefore, the results obtained from MRI scans may lack specificity, and the diagnosis is mostly determined by examining pathological evidence. The diagnosis relies on pathological observations.

MRI plays an important role in auxiliary diagnosis. The use of contrast enhancement contributes to diagnostic confidence and facilitates communication with patients. The MRI is the most effective method due to its extensive field of view, allowing for a comprehensive evaluation of the tumor’s spread inside the pelvic, intracardiac, and intravascular systems. Ultrasound dynamic observation may accurately demonstrate the correlation between the endovascular lesion and the vascular wall, therefore addressing the preoperative adhesion assessment requirements of medical professionals. The combination of MRI and ultrasound yields superior efficacy in diagnosing and identifying the IVL. When IVL invades the parametrial blood vessels, it is often misdiagnosed as broad ligament fibroids or ovarian tumors. Hence, it is imperative to prioritize individuals whose color ultrasound results indicate broad ligament lesions. Meanwhile pelvic MRI should be actively performed to improve the preoperative diagnosis rate, evaluate the degree of disease progression, and ensure adequate preoperative preparation (20).

The tumor appears “worm-like” on macroscopic examination, while histology and immunohistochemistry indicate the presence of benign desmin- and α-SMA-positive smooth muscle cells, together with varying expression of estrogen and progesterone receptors (19). Pathological and imaging studies reveal that the tumor’s base frequently has a connection to the uterine wall. HOXA13 may serve as a biomarker to distinguish between IVL and uterine myomas using RNA sequencing (21). However, additional research is urgently required to determine certain tumor biomarkers.

There is currently no agreement on the most effective surgical approach. Previous studies have indicated that intravenous leiomyomatosis can be classified into four stages, which aid in surgical treatment decisions (2, 3). Following complete tumor removal, the prognoses of these patients appeared more favorable. All patients with stage I tumors limited to the pelvis received oophorectomy and hysterectomy as part of their IVL treatment. However, research indicates that for certain stage I patients who are young women (≤40 years old), it is possible to preserve fertility by undergoing tumor excision alone as the main treatment. Hysterectomy or BSO (bilateral salpingo-oophorectomy) is not necessary in these cases (7). All patients graded stage II or above received total tumor resection, abdominal hysterectomy, and bilateral salpingo-oophorectomy. For stage III and IV patients, where the tumor progressed up the renal vein, resection was difficult without cardiopulmonary bypass, which was applied to avoid massive hemorrhage during tumor resection (2). Where the tumor progressed to the renal vein, surgery included incision of the inferior vena cava and removal of the tumor thrombus. As estrogen receptors are present in the tumor, in addition to surgery, it is possible to administer anti-estrogens such as tamoxifen if resection proves challenging or to prevent recurrence in the case of incomplete resection (22).

Parauterine involvement was an independent risk factor for increased intraoperative bleeding (20). Gao and Qu (20) demonstrated that age (<45 year) and surgical type (myomectomy) might be associated with disease recurrence. A few risk factors of recurrence are identified such as initial voluminous mass (>7 cm), impairment of broad ligament or failure of total surgical resection, as has been observed by Yu et al. (23). Studies have shown that the probability of IVL recurrence can exceed 30% (24), highlighting the importance of postoperative monitoring. Regarding postoperative follow-up, no consensus exists. Ma et al. (2) has suggested that patients were followed up every 6 months after surgery, and at each follow-up appointment received chest and abdominal CT and gynecological ultrasound and echocardiography again. Wang et al. (25) has recommended physical examination, echocardiography, pelvic ultrasound, and CTV examination of the IVC were performed at 3, 6, and 12 months after surgery, followed by annual to monitor recurrence risk. The primary endpoint of follow-up was complications and death, and the secondary endpoint was local recurrence (25). If a new mass was detected or the remnant tumor was enlarged, we considered the tumor recurred (2).

## Conclusion

Intrapelvic IVL is sometimes mistaken as a common benign uterine leiomyoma despite the fact that IVL tumors tend to be bigger. The MRI reveals a tumor that presents as a combination of solid and cystic lesions, showing intense and varied enhancement. It exhibits a high signal on diffusion-weighted imaging (DWI) and does not have a low apparent diffusion coefficient (ADC) value. In the present case, the base of the tumor was connected to the uterine wall and had a worm-like appearance. The diagnosis of intrapelvic IVL was confirmed by pathological examination.

## Data availability statement

The original contributions presented in the study are included in the article/supplementary material, further inquiries can be directed to the corresponding author.

## Ethics statement

The studies involving humans were approved by Medical Ethics Committee of Jinan Maternity and Child Care Hospital. The studies were conducted in accordance with the local legislation and institutional requirements. The human samples used in this study were acquired from Tumor tissue is from the operating theater. Pathological section is from department of pathology. Written informed consent for participation was not required from the participants or the participants’ legal guardians/next of kin in accordance with the national legislation and institutional requirements. Written informed consent was obtained from the patient(s) for the publication of any potentially identifiable images or data included in this article. Written informed consent was obtained from the patient(s) for the publication of this case report.

## Author contributions

BG: Writing – original draft. SZ: Investigation, Resources, Writing – original draft. JL: Resources, Writing – original draft. XW: Methodology, Writing – review & editing.
